# Estimating the effect of a rifampicin resistant tuberculosis diagnosis by the Xpert MTB/RIF assay on two-year mortality

**DOI:** 10.1371/journal.pgph.0001989

**Published:** 2023-09-01

**Authors:** Elise De Vos, Daniel Westreich, Lesley Scott, Yara Voss de Lima, Wendy Stevens, Cindy Hayes, Pedro da Silva, Annelies Van Rie

**Affiliations:** 1 University of Antwerp, Antwerp, Belgium; 2 University of North Carolina, Chapel Hill, NC, United States of America; 3 University of the Witwatersrand, Johannesburg, South Africa; 4 Instituto Nacional de Saúde, Maputo, Mozambique; 5 National Health Laboratory Service, Johannesburg, South Africa; 6 National Health Laboratory Services, Port Elizabeth, South Africa; Corporacion Universitaria Remington, COLOMBIA

## Abstract

Studies assessing patient-centred outcomes of novel rifampicin resistant tuberculosis (RR-TB) diagnostics are rare and mostly apply conventional methods which may not adequately address biases. Even though the Xpert MTB/RIF molecular assay was endorsed a decade ago for simultaneous diagnosis of tuberculosis and RR-TB, the impact of the assay on mortality among people with RR-TB has not yet been assessed. We analysed data of an observational prospective cohort study (EXIT-RIF) performed in South Africa. We applied a causal inference approach using inverse odds of sampling weights to rectify survivor bias and selection bias caused by differing screening guidelines. We also adjusted for confounding using a marginal structural model with inverse probability of treatment weights. We estimated the total effect of an RR-TB diagnosis made by the Xpert assay versus the pre-Xpert diagnostic algorithm (entailing a targeted Line Probe Assay (LPA) among TB-confirmed patients) on two-year mortality and we assessed mediation by RR-treatment initiation. Of the 749 patients diagnosed with RR-TB [247 (33%) by the pre-Xpert diagnostic algorithm and 502 (67%) by the Xpert assay], 42.7% died. Of these, 364 (48.6%) patients died in the pre-Xpert group and 200 (39.8%) in the Xpert group. People diagnosed with RR-TB by the Xpert assay had a higher odds of RR-TB treatment initiation compared to those diagnosed by the targeted LPA-based diagnostic process (OR 2.79; 95%CI 2.19–3.56). Receiving an RR-TB diagnosis by Xpert resulted in a 28% reduction in the odds of mortality within 2 years after presentation to the clinic (OR_CI_ 0.72; 95%CI 0.53–0.99). Causal mediation analysis suggests that the higher rate of RR-TB treatment initiation in people diagnosed by the Xpert assay explains the effect of Xpert on 2-year mortality [natural indirect effect odds ratio 0.90 (95%CI 0.85–0.96). By using causal inference methods in combination with high quality observational data, we could demonstrate that the introduction of the Xpert assay caused a 28% reduction in 2-year odds of mortality of RR-TB. This finding highlights the need for advocacy for a worldwide roll-out of rapid molecular tests. Because the effect is mainly caused by increased RR-TB treatment initiation, health care systems should also ensure timely initiation of effective treatment upon an RR-TB diagnosis.

## Introduction

Drug resistant tuberculosis (TB) is an important public health concern. Globally, nearly half a million cases of rifampicin-resistant TB (RR-TB) occurred in 2020 [1]. As part of the End TB strategy, the WHO advocates for intensified research and innovation through discovery, development, and rapid implementation of new diagnostic tools [2]. In the past decade, the pace of innovations in TB diagnostics has been rapid due to endorsement of cartridge-based molecular assays for the diagnosis of TB and rifampicin resistance (e.g. Xpert MTB/RIF (Xpert), Xpert MTB/RIF Ultra, TrueNat) and line probe assays (LPA) for diagnosis of resistance to isoniazid and rifampicin (GenoType MTBDR*plus* [3]), pyrazinamide (Genoscholar PZA-TB II LPA [4]), or fluoroquinolones and aminoglycosides (GenoType MTBDR*sl*, Xpert MTB/XDR [3]). A high pace of innovations is expected to continue as targeted sequencing, whole genome sequencing, and microtiter plates for phenotypic drug susceptibility testing (pDST) may be implemented in routine care in the near future [5]. Determining the effectiveness of new diagnostic tools will thus remain an important focus of TB research.

Policy makers increasingly (and rightfully) demand evidence of the effect of novel diagnostics on patient-centred outcomes. Most diagnostic studies focus on diagnostic accuracy such as sensitivity and specificity [6]. Unfortunately, increased test accuracy does not always translate into improved patient care [7]. For example, while many studies demonstrate high diagnostic accuracy of the Xpert assay [8,9] and improved time to diagnosis after its introduction [10–12], there was insufficient evidence for its impact on all-cause mortality [13].

The few studies that assessed patient-centred outcomes of novel TB diagnostics have mostly applied conventional methods (such as regression analysis) to estimate the association between the introduction of a new diagnostic and mortality [14–17]. Estimates from these conventional methods for observational data analysis are prone to bias due to (1) failure to take into account the selection bias that is introduced when including only RR-TB patients, as different diagnostic algorithms may yield populations of people diagnosed with RR-TB that are not comparable; (2) inadequate control for confounding; and (3) control for variables that should not be controlled for such as mediators or colliders [15,18–21]. In this study, we used modern epidemiologic methods that aim to overcome these biases when using observational data to assess the effect of the implementation of the Xpert assay versus the pre-Xpert diagnostic algorithm on mortality among people diagnosed with RR-TB in South Africa.

## Methods

### Study design

To evaluate the effect of the roll-out of the Xpert assay in South Africa on two-year all-cause mortality among people with RR-TB, we analysed data from an observational cohort study “Evaluating the Xpert Impact on Tuberculosis-RIFampicin Resistance” (EXIT-RIF). The study took place from Jan 2012 to Dec 2013, during the phased implementation of the Xpert assay in three provinces of South Africa (Free State, Gauteng and Eastern Cape).

### Study setting

In South Africa, diagnosis of drug resistant TB changed in 2011 with the use of the Xpert assay. Prior to the implementation of the Xpert assay, South Africa employed a centralised targeted LPA based RR-TB diagnosis. The initial diagnostic for people presenting with symptoms or signs of TB was sputum smear microscopy. If smear microscopy was positive, a first-line LPA assay (GenoType MTBDR*plus*) or pDST was requested for patients believed to be at increased risk for multidrug resistant TB (MDR-TB) given their history of TB treatment or contact with an RR-TB patient. If sputum smear microscopy was negative, a sputum culture for LPA or pDST was requested in people living with HIV [22]. Starting in 2011, the South African National Department of Health replaced centralised targeted LPA based RR-TB diagnosis with a decentralised Xpert assay as the initial diagnostic for TB [23]. Access to Xpert was rolled out during a 2–3 years phased implementation [24]. Because the Xpert assay simultaneously and accurately detects rifampicin resistance [25,26], the Xpert assay was also the initial diagnostic for RR-TB.

At the time of the study, all patients with RR-TB were initiated on a standardised treatment regimen of pyrazinamide, an aminoglycoside (amikacin or kanamycin), a fluoroquinolone, ethionamide and either cycloserine or PAS [27], as the present study predates the 2022 WHO guidelines [28]. Upon availability of DST results, guidelines recommended that treatment is individualised and contains at least 5 effective drugs.

### Study population

The National Health Laboratory Service (NHLS) database was screened monthly to identify all RR-TB diagnoses made at any health facility in the three provinces. All adults (≥18 years) diagnosed with RR-TB were eligible if not on treatment at the time of sputum sample collection. Patients were enrolled at the time of their RR-TB diagnosis by Xpert or LPA irrespective of whether they subsequently returned for RR-TB treatment or not, for example due to loss to follow-up (LTFU) or death.

### Data collection

Eligible patients (or next of kin) were interviewed by phone to collect clinical and socio-demographic data and to ascertain 2 year survival status. Healthcare providers were also interviewed by phone to document patient management such as treatment, additional diagnostics requested and patient follow-up by smear and cultures. All clinical information collected by phone was cross-referenced and verified through file reviews performed at all healthcare facilities where participants had received care for TB, HIV or any other condition between TB diagnosis and end of treatment, LTFU, or death. The NHLS Laboratory Information System was reviewed to collect results of TB diagnostics and HIV-related laboratory assays. The district TB register, individual patient medical files, and the national death register were reviewed to identify all deaths and to collect data on 2 year-survival status. All participants in whom no evidence of death was recorded were followed up by phone (answered by the participant or their next of kin), even if the patient had been lost to RR-TB care.

### Ethics

Ethical approval was obtained from the Human Research Ethics Committee of the University of the Witwatersrand in South Africa and the Institutional Review Board of the University of North Carolina at Chapel Hill in the United States. Approval of study activities was obtained from relevant health authorities. Participants gave verbal consent by phone (recorded). Waiver of consent was obtained for patients who had died or were LTFU from TB care prior to study enrolment and could not be contacted despite multiple attempts.

### Analysis

We described the distribution of participants’ baseline characteristics and the proportion or median (and interquartile range (IQR)) values of relevant variables in the pre-Xpert and Xpert period.

We framed our study question and analysis using a "target trial" approach [29–32]. The hypothetical randomized clinical trial (RCT), would randomize people who present with symptoms or signs of TB to either the pre-Xpert procedures for TB diagnosis including sputum smear microscopy and targeted LPA based RR-TB diagnosis or the intervention which consists of the Xpert assay for simultaneous diagnosis of TB and RR-TB. All trial participants diagnosed with RR-TB would be followed up for mortality in the two years after the diagnosis of RR-TB. To identify the biases that may have occurred by using observational data and to identify the covariates that should be included in the analysis to overcome these biases, we drew Directed Acyclic Graphs (DAGs). DAGs are a graphical representations of the relationships between exposure, outcome and variables associated with the exposure and outcome under investigation [20].

We performed causal inference analyses to reduce the bias introduced by the observational study design. We rectified selection bias using inverse odds of sampling weights (IOSWs) and confounding using a marginal structural model (MSM) with inverse probability of treatment weights (IPTWs). We estimated the total effect of a diagnosis of RR-TB by Xpert on 2-year mortality and performed causal mediation analysis to disentangle the causal pathway. Given zero missingness in exposure or outcome data, and limited missingness (<11%) in all covariates, we conducted a complete case analysis.

#### Reducing selection bias due to differential LTFU by reweighting participants in the pre-Xpert group to the Xpert group using inverse odds of sampling weights (IOSWs) for each individual

**Fig 1:**
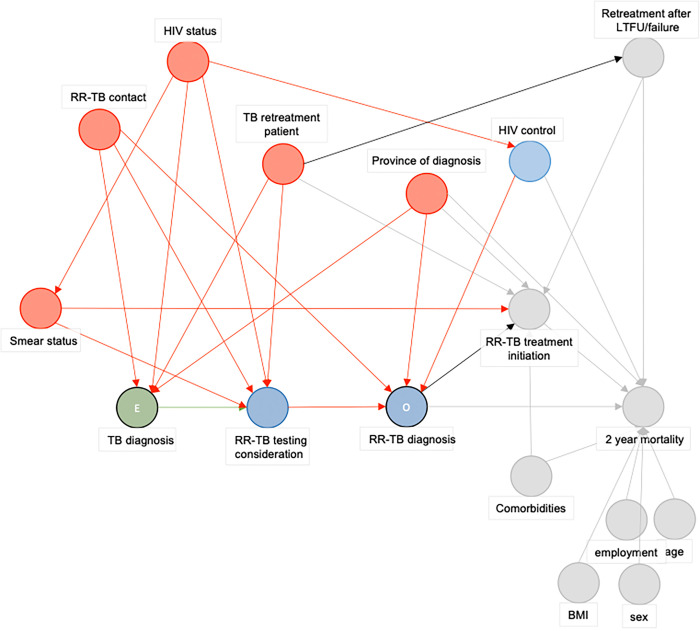
Directed Acyclic Graph to guide the selection of variables included in the inverse odds of sampling weights analysis. Abbreviations: E: Exposure, HIV: Human immunideficiency virus, LTFU: Loss to follow-up, O: Outcome, RR-TB: Rifampicin-resistant tuberculosis. Green circle represents the exposure, blue circles represent (ancestors of) the outcome. Red circles are ancestral variables of both the exposure and the outcome, and are in this analysis variables on which selection bias occurred in the targeted LPA based RR-TB diagnostic algorithm. Grey circles are variables not of importance in this exposure-outcome relationship. Green paths are causal pathways, red paths are biasing pathways.

In the observational EXIT-RIF study, participants were captured at RR-TB diagnosis. This differs from the hypothetical RCT, where all patients with signs and symptoms of TB would be included and randomized to the pre-Xpert or Xpert approach to diagnose RR-TB. The simultaneous detection of both TB and RR-TB with high sensitivity (94.4%) by the Xpert assay results in diagnosis of almost all RR-TB cases among people presenting with signs and symptoms of TB [33,34]. Thus, the population diagnosed by Xpert was similar to the population in the experimental arm of the hypothetical RCT. Therefore, we assumed that participants diagnosed with RR-TB by Xpert in the observational study represented an unbiased sample of the target population. In contrast, the two-step diagnostic process for RR-TB in the pre-Xpert arm most likely resulted in a biased sample of the target population of the hypothetical RCT, for three reasons. First, patients may never have received a TB diagnosis due to the low sensitivity (67·1%) of the initial smear microscopy test [33,34]. Second, patients diagnosed with TB needed to be considered for RR-TB assessment by LPA. Third, TB-confirmed patients may subsequently have been lost to follow up or dead in the time period between the diagnosis of TB and the assessment for RR-TB by LPA. Consequently, those enrolled in the pre-Xpert study period are a biased sub-population of the population of interest. To correct for this selection bias, we implemented IOSW to reweight participants in the pre-Xpert group to the Xpert group based on variables selected by a DAG (Fig 1) [35]. In this DAG, the exposure is a TB diagnosis among patients presenting to the clinic and the outcome is an RR-TB diagnosis (Fig 1). According to the DAG, variables introducing selection bias were smear status, RR-TB contact, HIV status, TB retreatment, and the province of diagnosis.

**Fig 2:**
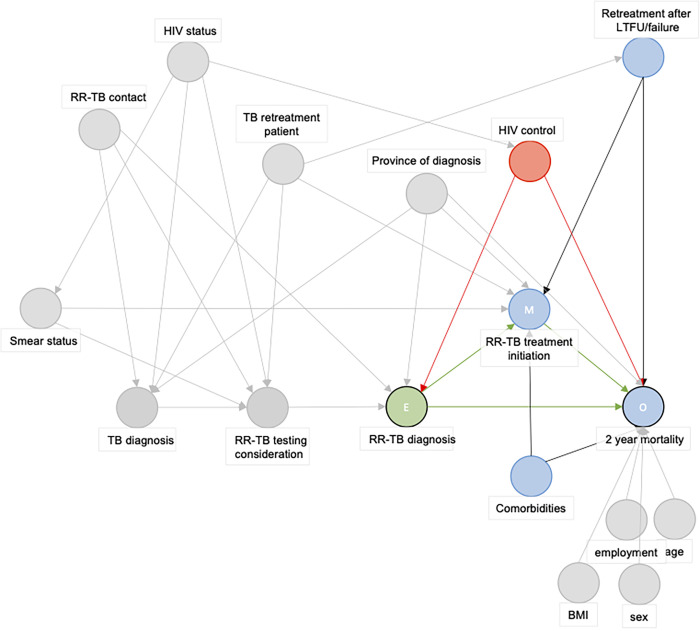
Directed Acyclic Graph used to identify the confounding paths of the exposure (RR-TB diagnosis by Xpert versus pre-Xpert targeted LPA-based RR-TB diagnostic algorithm)—outcome (two-year mortality) relationship. Abbreviations: E: Exposure, HIV: Human immunideficiency virus, LTFU: Loss to follow-up, M: Mediator, O: Outcome, RR-TB: Rifampicin-resistant tuberculosis. The green circle represents the exposure, blue circles represent (ancestors of) the outcome. Red circles are ancestral variables of both the exposure and the outcome and is a confounder in the exposure-outcome relationship. Grey circles are variables not of importance in this exposure-outcome relationship. Green paths are causal pathways, red paths are biasing pathways.

To estimate the individual weights (W_i_), the inverse of an individual’s conditional sampling odds was multiplied by the unconditional sampling odds. The inverse of an individual’s conditional sampling odds was defined as the inverse of the ratio of the individual’s probability of being a member of the pre-Xpert EXIT-RIF study population P(S_i_ = 1) as opposed to the Xpert EXIT-RIF population P(S_i_ = 0), conditional on their covariate values Z_i_ (Figs 1 and 2). The unconditional sampling odds were defined as the ratio of the (unconditional) probability of belonging to the pre-Xpert EXIT-RIF study population to the ratio of the (unconditional) probability of belonging to the Xpert EXIT-RIF population.


$$W_{i}=\left\{ \begin{array}{r} \frac{P(S_{i}=O|Z_{i})}{P(S_{i}=1|Z_{i})}X\frac{P(S_{i}=1)}{P(S_{i}=0)},S_{i}=1\\ 0,S_{i}=0 \end{array}\right.$$

#### Reduce confounding using a Marginal structural model (MSM) with Inverse probability of treatment weights (IPTW) to estimate the total effect of an RR-TB diagnosis by Xpert on 2-year mortality

Since the EXIT-RIF study was observational, the intervention, i.e., the use of Xpert to diagnose RR-TB was not randomized. This may have introduced confounding of the association between the diagnostic algorithm used for RR-TB diagnosis and 2-year mortality.

**Fig 3:**
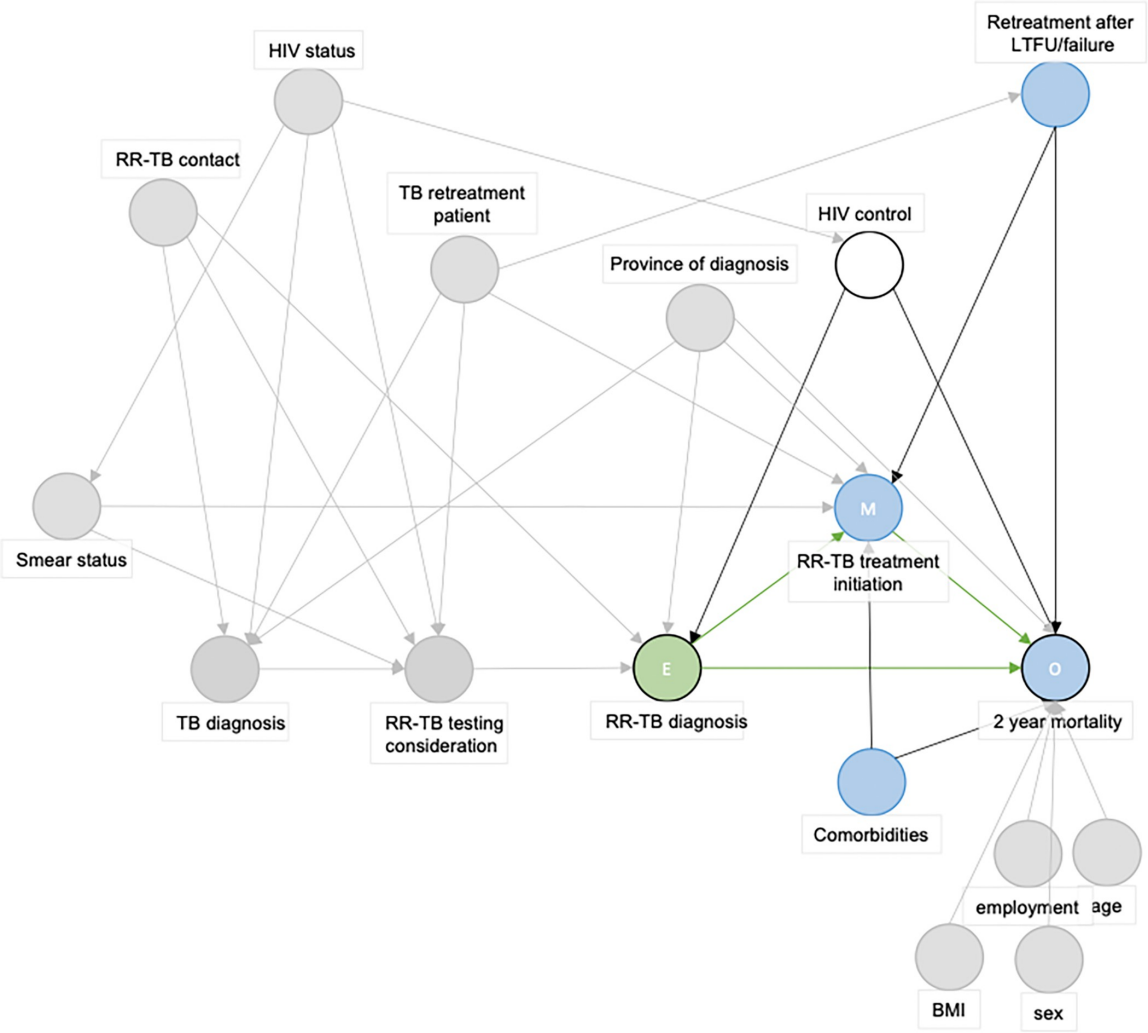
Directed Acyclic Graph used to guide the margninal structural model (MSM) assessing the (total) effect of the exposure (RR-TB diagnosis by Xpert versus pre-Xpert targeted LPA-based RR-TB diagnostic algorithm) on the outcome (two-year mortality) relationship. Abbreviations: E: Exposure, HIV: Human immunideficiency virus, LTFU: Loss to follow-up, M: Mediator, O: Outcome, RR-TB: Rifampicin-resistant tuberculosis. The green circle represents the exposure, blue circles represent (ancestors of) the outcome. White cirkle represents an adjusted variable. Grey circles are variables not of importance in this exposure-outcome relationship. Green paths are causal pathways, red paths are biasing pathways.

We therefore drew a DAG where the exposure is an RR-TB diagnosis by Xpert or pre-Xpert diagnostic algorithm and the outcome is 2-year mortality. The DAG identified that the level of HIV control was the only confounding variable and showed that it is not required to control for age, employment, BMI, or sex in the analysis (Figs 1–3).

To reduce potential confounding introduced in the observational study, we applied inverse probability of treatment weights (IPTW). IPTW reweights each individual in the population by the inverse of the conditional probability (conditional on the confounder HIV control) of receiving the ‘treatment’ [being diagnosed with RR-TB by the pre-Xpert algorithm or by the intervention (Xpert)]. IPTW thus creates a hypothetical pseudo-population twice as large as the observed study population, where each individual appears both as exposed and unexposed. By doing so, the marginal risk of experiencing the outcome (2-year mortality) under a diagnosis (by the pre-Xpert algorithm or by Xpert) is independent of the actual method of diagnosis received in the observational study [20,36–40] (Fig 3). This process is a key component of the counterfactual framework and helps to ensure that the conditional exchangeability assumption for causal inference is met [36–39].

After applying both IOSWs and IPTWs, we estimated the OR of RR-TB treatment initiation in the Xpert population versus the pre-Xpert population and the total effect of having a diagnosis made by Xpert on (all-cause) 2-year mortality. Properties of the different weights can be found in the S1 Text.

#### Causal mediation analysis

We hypothesized that the proportion of people who initiate RR-TB treatment may be higher among people diagnosed with RR-TB by the Xpert assay and that this mediator could explain the effect of the Xpert assay on RR-TB mortality (Fig 3). To assess whether the effect of implementation of Xpert on mortality is explained by higher rates of RR-TB treatment initiation or other factors, we performed a causal mediation analysis [19].

We performed the analysis using a weighting-based approach (ratio of mediator probability weighting) to create a pseudo-population. In this hypothetical population, each individual has an outcome (2-year mortality) and mediator variable (whether RR-TB treatment was initiated or not) as was observed in the data (the individual’s true outcome and mediator), as well as a counterfactual outcome and mediator value, which would have occurred if the individual had been diagnosed with the diagnostic algorithm (pre-Xpert or Xpert) other than the one they were diagnosed with in the observational study.

Using this ‘pseudo-population’ dataset, a natural effect model was then applied using the R package Medflex, to estimate the natural indirect effect (NIE) [41], which is the effect of the mediator (RR-TB treatment initiation) when the exposure is set as an RR-TB diagnosis by Xpert (S1 Fig).

## Results

The EXIT-RIF observational cohort study (Jan 2012- Dec 2013) enrolled 749 RR-TB patients of which 247 (33%) were diagnosed with RR-TB by the pre-Xpert diagnostic process and 502 (67%) by the Xpert assay. Patients were diagnosed in three South African provinces: Gauteng (n = 236, 32%), Free State (n = 192, 26%), and Eastern Cape (n = 321, 43%) (Table 1). Because of the phased implementation of the Xpert assay, the proportion of patients diagnosed with RR-TB by Xpert increased from 49.7% (n = 240) in 2012 to 98.5% (n = 262) in 2013. Almost all patients (99.1%) had pulmonary RR-TB, smear microscopy status was positive in 45, mean BMI was 20.2kg/m^2^ (SD 6.2). Half (52.2%) of all patients had a history of TB treatment, and 17.7% (n = 133) patients reported another predictive factor for RR-TB [close contact with an MDR-TB patient (n = 111) or working at a healthcare facility (n = 22)]. Most (78%) patients were living with HIV, of whom only one in three (33%) were on ART.

**Table 1:** Study population.

	All		Pre-Xpert*		Xpert	
	N = 749		N = 247		N = 502	
Sex = Female (%)	367	0.48	126	51	241	48
age (mean (SD))	38.19	11.28	37.93	10.53	38.31	11.65
BMI (mean (SD))	20.20	6.19	20.73	5.64	19.89	6.49
Risk factors(%)						
none reported	616	82.2	196	79.4	420	83.7
Family/friend with MDR-TB	111	14.8	43	17.4	68	13.5
Worked at clinic/hospital	22	2.9	8	3.2	14	2.8
Province (%)						
Gauteng	236	31.5	81	32.8	155	30.9
Free State	192	25.6	51	20.6	141	28.1
Western Cape	321	42.9	115	46.6	206	41.0
Employment (%)						
No	351	46.9	116	47	235	46.8
Yes	117	15.6	40	16.2	77	15.3
other	281	37.5	91	36,8	190	37,8
Substance abuse (%)	26	3.5	5	2	21	4.2
Diabetes Mellitus (%)	28	3.7	10	4	18	3.6
Mental Health Concerns (%)	31	4.1	10	4	21	4.2
HIV status (%)						
negative	144	19.2	45	18.2	99	19.7
positive	581	77.6	194	78.5	387	77.1
unknown	24	3.2	8	3.2	16	3.2
CD4 (median [IQR])	133.00	51.50, 276.50	128.00	51.00, 307.50	135.50	52.00, 272.25
Baseline log viral load (mean (SD))	4.56	1.49	4.33	1.55	4.75	1.43
Baseline ART status (%)						
ART interrupted	42	7.2	13	6.7	29	7.5
ART naïve	187	32.2	62	32	125	32.3
on ART at diagnosis	193	33.2	62	32	131	33.9
Unknown	159	27.4	57	29.4	102	26.4
TB type (%)						
Pulmonary TB (PTB)	689	92.0	223	90.3	466	92.8
Extrapulmonary TB (EPTB)	7	0.9	3	1.2	4	0.8
PTB+EPTB	53	7.1	21	8.5	32	6.4
Newly diagnosed (%)						
Newly diagnosed	303	40.5	80	32.4	223	44.4
Retreatment	391	52.2	148	59.9	243	48.4
Unknown	55	7.3	19	7.7	36	7.2
Retreatment type (%)			n = 148		n = 242	
Cured	21	54.3	69	46.6	143	59.1
Defaulted	87	22.36	43	29.1	44	18.2
Treatment failure	18	4.6	10	6.8	8	3.3
Other	73	18.7	26	17.6	47	19.4
Smear status (%)						
Negative	333	44.5	96	38.9	237	47.2
No smear info	80	10.7	17	6.9	63	12.5
Positive	336	44.9	134	54.3	202	40.2

* targeted LPA based RR-TB diagnostic algorithm.

Compared to patients diagnosed by Xpert, more patients diagnosed in the pre-Xpert period were smear positive (47% vs 38%), had been treated for TB before (60% vs 48%) with default (29% vs 18%) or failure (7% vs 3%) during their prior treatment episode. The distribution of province and ART status also differed between the two groups. Crude 2-year mortality was 48.6% in the pre-Xpert group and 39.8% in the Xpert period.

When applying IOSW, IPTW and MSM for causal inference, we found that receiving an RR-TB diagnosis by Xpert reduced 2-year mortality (OR_CI_ 0.72; 95%CI 0.53–0.99) (Table 2). This represents an estimated 28% reduction in 2-year mortality odds. As expected, people diagnosed with RR-TB by Xpert had a higher odds of RR-TB treatment initiation compared to those diagnosed by the targeted LPA-based RR-TB diagnostic algorithm used in the pre-Xpert period (OR 2.79; 95%CI 2.19–3.56). In the mediation analysis, the natural indirect effect odds ratio (OR_NIE_, the effect that runs through the mediator RR-TB treatment initiation) was 0.90 (95%CI 0.85–0.96). The estimate of the natural direct effect OR (OR_NDE_., the effect of a diagnosis by Xpert without any effect of Xpert on RR-TB treatment initiation) was lower (0.80) but the 95% CI crossed 1 (95%CI 0.59–1.10). These results suggest that the higher rate of treatment initiation modifies the effect of Xpert on 2year mortality, and that the effect of Xpert on 2-year mortality compared to the pre-Xpert diagnostic algorithm may be due to higher rates of RR-TB treatment initiation.

**Table 2:** Estimate of the total implementation of the Xpert MTB/RIF assay on 2-year mortality (crude and adjusted for biases using causal inference methods) and the indirect effect when including initiation of treatment for rifampicin resistant tuberculosis as a mediator.

Estimate	OR	95% CI
**Total effect estimated by causal inference methods**	0.72	0.53–0.99
**Natural indirect effect (NIE)**	0.90	0.85–0.96

## Discussion

In this study, we found that use of the Xpert assay causes an increase in odds of starting RR-TB treatment (OR 2.79 95%CI 2.19–3.56) and a reduction in the 2-year odds of death (OR_CI_ 0.72; 95%CI 0.53–0.99) among people who presented to a health facility with symptoms of TB due to RR-TB. Moreover, the presence of a natural indirect effect (OR_NIE_ 0.90; 95%CI 0.85–0.96) suggests that the total effect of Xpert on mortality is mostly due to an effect of the Xpert assay on RR-TB treatment initiation rather than a direct effect of the diagnostic itself. Taken together, we show that, among people with signs and symptoms of TB (due to RR-TB) presenting to a clinic, the use of the Xpert assay causes a reduction in mortality, mainly through an increase in RR-TB treatment initiation.

We could not compare our finding of an effect of Xpert on mortality to reports in other studies, as no previous observational study or clinical trial assessed the effect of the use of Xpert on treatment outcomes in people suffering from RR-TB. This is in stark contrast to the many studies that assessed the impact of using Xpert on treatment outcomes in patients presenting with symptoms of (drug susceptible or any) TB [42,43]. The significant NIE we observed indicates that the effect of Xpert acts through initiation of RR-TB treatment, and supports the findings of a retrospective study in Rwanda where a delay of ≥100 days was associated with a 2.58 higher odds of dying [21]. We found one trial assessing the impact of Xpert on time to culture conversion, not taking into account the selection bias introduced by only including RR-TB patients [15]. A major strength of our study is the application of the causal inference framework to emulate a hypothetical clinical trial using data from an observational or semi-experimental study [44,45]. This approach allowed us to estimate the ‘unbiased’ effect of the use of Xpert RR-TB on 2-year mortality in people who present with symptoms of TB due to the presence of RR-TB. To achieve this, we combined IOSWs to overcome the selection bias introduced by the observational pre-Xpert group with IPTWs to correct for the confounding bias introduced in absence of randomization [36,38,46–48]. The use of causal inference methods allowed us to correctly frame the question, as highlighted by Chani et al. [49]: ‘Among people presenting to the clinic with signs and symptoms; to what extent does the use of Xpert cause a reduction in 2-year mortality among patients with RR-TB?’. While the use of causal inference methodology has already found its way in HIV research [50–52], its application is novel in the field of TB and DR-TB [53–56].

Another important strength of our study relates to the quality of the observational data used to emulate the hypothetical RCT. The EXIT-RIF study was a prospective cohort study aimed at determining the association between the implementation of Xpert and mortality in people diagnosed with RR-TB. The use a of centralized laboratory system to determine eligibility allowed the study to include all patients diagnosed with RR-TB in three provinces, not only those who present for RR-TB care at selected study sites which is common in many studies of RR-TB. The detailed collection of data on 2-year mortality though multiple sources resulted in complete data which allowed us to accurately assess the effect of Xpert on 2-year mortality, even for those lost to RR-TB care.

A limitation of the study is that we did not account for missing data in covariates. We do not believe that this greatly biased the results as none of the variables included in the analyses had missingness greater than 11%. Moreover, while Xpert’s sensitivity is excellent (±94.4% [33,34]), it remains below 100% and could have introduced some bias in our analysis, as we assumed that the Xpert population is an unbiased sample of the target population. Another limitation is that we treated RR-TB treatment initiation and mortality as binary variables. Because RR-TB treatment initiation was treated as a binary variable (initiated RR-TB treatment or not) rather than a continuous variable (time from diagnosis to treatment initiation), the effect is likely to be an underestimation as earlier treatment initiation is not distinguished from later initiation. Methods for causal (mediation) analyses using time-to-event data exist [57,58] but their use was beyond the scope of our analysis. Finally, the observational EXIT-RIF study took place in South Africa, a setting which is not generalizable to all high RR-TB burden settings. The 28% reduction in mortality odds is likely an underestimation of the effect that can be expected in many countries with a high RR-TB burden where LPA has not yet been implemented, as opposed to South Africa. In contrast, in settings where the prevalence of resistance to fluoroquinolones and new drugs such as Bedaquiline is higher than in South Africa, the effect on mortality of a rapid molecular test to simultaneously diagnose tuberculosis and rifampicin resistance may be lower than in the current study.

In conclusion, using causal inference methods in combination with high quality observational data, we provide a robust answer to a research question of high policy and patient relevance: ‘does the routine use of a new rapid molecular assay result in a reduction in RR-TB mortality?’. Our analysis provides confirmatory evidence that the use of Xpert reduces 2-year mortality among people presenting to the clinic with signs and symptoms of TB due to RR-TB. This highlights the need for advocacy for a continued global roll-out of rapid molecular tests to diagnose TB drug resistance. Furthermore, as mediation analysis showed the effect is mainly due to a higher probability of RR-TB treatment initiation, health care systems should ensure timely initiation of treatment regimens upon diagnosis of RR-TB.

## Supporting information

S1 Fig Graphic representation of the total effect (TE), Natural Direct Effect (NDE) and Natural Indirect Effect (NIE). The Expected (E) value of the outcome (2-year mortality) (Y) under a certain exposure status [(1) being exposed to a Xpert diagnosis, (0) having a diagnosis made under SOC], and certain mediator value (RR-TB treatment) under a given exposure status [M(1) being the mediator value when exposed, M(0) being the mediator value when unexposed].
(DOCX)

S1 Text Properties of the Inverse Odds of Sampling weights, Inverse probability of treatment weights. (DOCX)
