# Supplementary material for: Estimating the effect of a rifampicin resistant tuberculosis diagnosis by the Xpert MTB/RIF assay on two-year mortality
Source: PLOS Glob Public Health. 2023 Sep 1;3(9):e0001989. doi: 10.1371/journal.pgph.0001989 (PMC10473529; doi:10.1371/journal.pgph.0001989)
Supplement: S1 Fig — The Expected (E) value of the outcome (2-year mortality) (Y) under a certain exposure status [(1) being exposed to a Xpert diagnosis, (0) having a diagnosis made under SOC], and certain mediator value (RR-TB treatment) under a given exposure status [M(1) being the mediator value when exposed, M(0) being the mediator value when unexposed]. (DOCX) [file pgph.0001989.s001.docx]

**S1 Fig. Graphic representation of the total effect (TE), Natural Direct Effect (NDE) and Natural Indirect Effect (NIE).**

The Expected (E) value of the outcome (2-year mortality) (Y) under a certain exposure status [(1) being exposed to a Xpert diagnosis, (0) having a diagnosis made under SOC], and certain mediator value (RR-TB treatment) under a given exposure status [M(1) being the mediator value when exposed, M(0) being the mediator value when unexposed]

Abbreviations: RR-TB: rifampicin-resistant tuberculosis; SOC: standard of care
