# Supplementary material for: Estimating the effect of a rifampicin resistant tuberculosis diagnosis by the Xpert MTB/RIF assay on two-year mortality
Source: PLOS Glob Public Health. 2023 Sep 1;3(9):e0001989. doi: 10.1371/journal.pgph.0001989 (PMC10473529; doi:10.1371/journal.pgph.0001989)
Supplement: S1 Text — (DOCX) [file pgph.0001989.s002.docx]

**S1 Text. Properties of the Inverse Odds of Sampling weights, Inverse probability of treatment weights**

Inverse odds of Sampling weights: Mean,sd (pre-Xpert group) 1.22, 0.57

Mean,sd (pre-Xpert group) 1, 0

Inverse Probability of Treatment Weights mean, sd (overall) 2.00, 0.83
